# Over-expression of AhR (aryl hydrocarbon receptor) induces neural differentiation of Neuro2a cells: neurotoxicology study

**DOI:** 10.1186/1476-069X-5-24

**Published:** 2006-09-07

**Authors:** Eiichi Akahoshi, Seiko Yoshimura, Mitsuko Ishihara-Sugano

**Affiliations:** 1 Environmental Technology Laboratory, Corporate Research & Development Center, Toshiba Corporation, 1 Komukai-Toshiba cho, Saiwai-ku, Kawasaki 212–8582, Japan

## Abstract

**Background:**

Dioxins and related compounds are suspected of causing neurological disruption in human and experimental animal offspring following perinatal exposure during development and growth. The molecular mechanism(s) of the actions in the brain, however, have not been fully investigated. A major participant in the process of the dioxin-toxicity is the dioxin receptor, namely the aryl hydrocarbon receptor (AhR). AhR regulates the transcription of diverse genes through binding to the xenobiotic-responsive element (XRE). Since the AhR has also been detected in various regions of the brain, the AhR may play a key role in the developmental neurotoxicity of dioxins. This study focused on the effect of AhR activation in the developing neuron.

**Methods:**

The influence of the AhR on the developing neuron was assessed using the Neuro2a-AhR transfectant. The undifferentiated murine neuroblastoma Neuro2a cell line (ATCC) was stably transfected with AhR cDNA and the established cell line was named N2a-Rα. The activation of exogenous AhR in N2a-Rα cells was confirmed using RNAi, with si-AhR suppressing the expression of exogenous AhR. The neurological properties of N2a-Rα based on AhR activation were evaluated by immunohistochemical analysis of cytoskeletal molecules and by RT-PCR analysis of mRNA expression of neurotransmitter-production related molecules, such as tyrosine hydroxylase (TH).

**Results:**

N2a-Rα cells exhibited constant activation of the exogenous AhR. CYP1A1, a typical XRE-regulated gene, mRNA was induced without the application of ligand to the culture medium. N2a-Rα cells exhibited two significant functional features. Morphologically, N2a-Rα cells bore spontaneous neurites exhibiting axon-like properties with the localization of NF-H. In addition, cdc42 expression was increased in comparison to the control cell line. The other is the catecholaminergic neuron-like property. N2a-Rα cells expressed tyrosine hydroxylase (TH) mRNA as a functional marker of catecholaminergic neurotransmitter production. Thus, exogenous AhR induced catecholaminergic differentiation in N2a-Rα cells.

**Conclusion:**

The excessive activation of AhR resulted in neural differentiation of Neuro2a cells. This result revealed that dioxins may affect the nervous system through the AhR-signaling pathway. Activated AhR may disrupt the strictly regulated brain formation with irregular differentiation occurring rather than cell death.

## Background

The aryl hydrocarbon receptor (AhR) is a ligand-activated transcription factor belonging to a basic helix-loop-helix/Per-Arnt-Sim (bHLH-PAS) family [1,2]. AhR is activated by a variety of environmental contaminants including polycyclic aromatic hydrocarbons (PAH), planar chlorinated biphenyls (PCB), and halogenated aromatic hydrocarbons (HAH) such as 2, 3, 7, 8, -tetrachlorodibenzo-*p*-dioxin (TCDD) [3,4]. Several major signal transduction pathways are mediated by the AhR, allowing dioxins to affect diverse biological processes [5,6].

The AhR is activated by binding to ligands, including dioxins, translocates from the cytoplasm to the nucleus and then binds to the consensus sequence known as XRE (xenobiotic responsive element) [7]. XREs are found in the promoter region of a variety of genes. Several phase I and phase II xenobiotic metabolizing enzymes, including cytochrome P-450 (CYP) 1A1, CYP1B1 and glutathione S-transferase (GST) possess multiple XRE sequences in the promoter region, and are the target genes for AhR following binding to the AhR ligands [8-11]. In addition to the xenobiotic or detoxification processes, the ligands of AhR, such as TCDD, also affect the expression of a number of genes involved in cell proliferation (TGF-β, IL-1β and PAI-2), cell cycle regulation (p27 and jun-B) [5,12-14] and inflammation. The AhR pathway may participate in the developmental toxication of dioxins during ontogenesis.

One of the dioxins, TCDD, can also influence the developing brain. After exposure to experimental animals, TCDD was detected in the brain and impaired their learning behavior. Perinatal exposure to TCDD has also been found to remarkably affect learning ability in rats and monkeys [15-17]. However the molecular mechanisms of the action of TCDD in the brain have not been fully elucidated.

The dioxin receptor, namely the AhR, is expressed in various regions of the brain [18,19] during the critical period of brain development [20,21]. In particular, it has been reported that maximal expression of AhR protein was observed during P3–10 [22]. This stage is considered to be the critical period for the growth and maturation of neuroblasts in the cerebellar granular cell layer. These findings imply that the AhR may be involved in the molecular processes of brain development, and may also be related to the developmental neurotoxicity of TCDD. However, it has been very difficult to directly examine the actual role of AhR in the pathway from TCDD to the function during brain development, because an experimental model has not yet been established to investigate AhR function in developing neurons.

The regulation of neurogenesis is key event for controlling brain development. To understand the regulatory mechanisms of neurogenesis, neuroblastoma is widely used as an experimental model. Neuroblastoma usually arises from the proliferation of neural crest-derived precursors resulting in defective differentiation *in vivo *[23]. Neuroblastoma cells proliferate as undifferentiated cells in standard cell culture, but they have the potential to become neuronal precursors [24,25]. Neuro2a cells are a murine neuroblastoma cell line that has proven to be a useful experimental model for studying some aspects of differentiation mechanism since its differentiation can be controlled by various conditions, such as low serum concentration, retinoic acid and cAMP [26,27]. In the present study, we transfected a neuroblastoma cell line, Neuro2a, with cloned AhR and examined the effect of over-expression of the AhR on these cells. This stable transfectant will be an appropriate experimental model for analyzing the relationships between AhR activation and developmental neurotoxicity.

## Methods

### Cell cultures

Neuro2a, a murine neuroblastoma cell line, was purchased from American Type Culture Collection, and was routinely grown in Dulbecco's modified Eagle's medium and medium F12 in a 1:1 ratio, supplemented with 10% fetal calf serum. Cells were grown at 37°C in a humidified atmosphere of 5% CO_2_/95% air.

### Construction of expression vectors

The coding sequence of AhR was obtained by RT-PCR from rat brain RNA with *Pyrobest *DNA polymerase (TAKARA BIO Inc., Shiga, Japan). PCR primers were designed to contain appropriate restriction sites for subcloning. The sequences of the primers were: 5'-CCCAAGCTTACCATGAGCAGCGGCGCCAACATCA-3' (*Hin*dIII) and 5'-CCGCTCGAGAGGAATCCGCTGGGTGTGATATCAG-3' (*Xho*I). After digestion with *Hin*dIII and *Xho*I, the PCR product was cloned into the mammalian expression vector pcDNA4/V5-His (Invitrogen Japan KK, Tokyo, Japan) digested with *Hin*dIII and *Xho*I. The resulting vector contained the coding sequence of AhR fused to the V5/His tag under the control of cytomegalovirus promoter. Correct insertion of the PCR product was verified by DNA sequencing.

### Stable introduction of AhR into the Neuro2a cell line

Neuro2a cells were maintained and grown in Dulbecco's modified Eagle's medium and medium F12 in a 1:1 ratio, supplemented with 10% fetal calf serum. The cells (8 × 10^4^) were transfected with 0.8 μg of constructed plasmid using Lipofectamine 2000 according to the manufacturer's instructions (Invitrogen Japan KK). Cells transfected with a vector without the insert were used as a control. After culturing for 72 h in normal medium, the cells were switched to medium supplemented with 250 μg/ml antibiotic zeocin (Invitrogen Japan KK). The medium was changed every 3 days during selection of viable cells. After 2 weeks, viable cells were isolated using cloning rings. The isolated clones were cultured in medium supplemented with 250 μg/ml zeocin for another 2 weeks and the viable clones were selected for further characterization.

### Reverse Transcriptase (RT)-PCR

Total RNA was isolated from cultured cells following a 2-day culture using RNeasy (Qiagen KK, Tokyo, Japan). Rat brain RNA was extracted from 8-week-old Sprague-Dawley rats. The rats were killed by cervical dislocation under ether anesthesia and the brains were dissected.Total RNA was extracted by Quick Prep Total RNA Extraction Kit (GE Healthcare Bio-Sciences Corp. NJ, USA). Total RNA of mouse brains were purchased from ZYAGEN laboratories (CA, USA). These RNAs were reverse-transcribed into cDNA using Superscript II reverse transcriptase (Invitrogen Japan KK). PCR was performed with *Ex Taq *polymerase (TAKARA BIO Inc.) using gene-specific primers. The primer sequences were as follows: AhR, 5'-CCGTCCATCCTGGAAATTCGAACC -3', 5'-CCTTCTTCATCCGTTAGCGGTCTC; CYP1A1, 5'-TTGCCCTTCATTGGTCACAT-3', 5'-GAGCAGCTCTTGGTCATCAT-3'; AhRR, 5'-CCCAAACTCAATTACTTAGCAGG-3', 5'-GCTTCCAGGGTAGGGCACACAGG-3'; GST, 5'-CCTGGCTGCAGCAGGGGTGGAG-3', 5'-CGGTTTTTGGTCCTGTCTTTTGC-3'; cdc42, 5'-GATACTGCAGGGCAAGAGGA -3', 5'-CAGGCACCCACTTTTCTTTC-3'; TH, 5'-CCACGGTGTACTGGTTCACT-3', 5'-GGCATAGTTCCTGAGCTTGT -3'; Nurr, 5'-CCAATCCGGCAATGACCAG-3', 5'-GTCAGCAAAGCCAGGGATCTTC-3'; Cyclophilin, 5'-GTCTCCTTCGAGCTGTTTGCAG-3', 5'-CCATCCAGCCATTCAGTCTTGG-3'. PCR products were electrophoresed on 3% Nusieve 3:1 agarose gel (TAKARABIO inc.) and then stained with ethidium bromide. The intensities ofthe bands were quantified using ImageJ software [28] and then normalized to those of cyclophilin.

### RNA interference

Two siRNAs, si-AhR and scramble, were synthesized (Qiagen KK) and transfected into the cells using RNAiFect according to the manufacturer's instructions (Qiagen KK). The siRNA sequences were: si-AhR, 5'-UCCCACAUCCGCAUGAUUATT-3', 5'-TTAGGGUGUAGGCGUACUAAU-3'; scramble, 5'-UACCCAUCAUGACCCUGAUTT-3', 5'-TTAUGGGUAGUACUGGGACUA-3'. Two days after transfection, total RNA was extracted from the cells and reverse-transcribed with Superscript II reverse transcriptase. The cDNAs were used in real-time PCR as templates for quantification.

### Real-Time PCR

AhR, CYP1A1 and cdc42 mRNA expression were quantified in AhR by real-time PCR. The primers used in real-time PCR were the same as those described above. Real-time PCR was carried out with DNA engine (Bio-Rad Japan, Tokyo, Japan) using the DyNAamo SYBR Green qPCR Kit (Finnzymes, Oy, Finland). The PCR conditions were: 95°C for 5 min, 40 cycles of 95°C for 10 s, 58°C for 10 s, and 72°C for 20 s with fluorescence measurements read during each cycle. The expression levels of AhR and CYP1A1 mRNAs were normalized to those of cyclophilin mRNA. The series of measurements was performed four times.

### Western Blot analysis

Tissues and cells were homogenized in phosphate buffered saline (PBS) and denatured with SDS. Total protein concentrations were determined by Bio-Rad Protein Assay (Bio Rad Laboratories Inc., USA). Proteins (30 μg/lane) were fractionated on 10% SDS-PAGE and transferred to PVDF membrane (Millipore MA, USA). Membranes were blocked with Block Ace (Dainippon Seiyaku, Osaka, Japan) and then incubated with an anti-AhR mouse monoclonal antibody (1:500; Affinity BioReagents, CO, USA). After incubation with the primary antibody, the membrane was washed three times with tris-buffered saline (TBS). The blots were subsequently exposed to an alkaline phosphatase-conjugated rabbit anti-mouse IgG (1:1000; Bio-Rad). The blots were visualized with BCIP-NBT substrate solution (Kirkegaard & Perry Laboratories, Inc. MD, USA).

### Immunofluorescence microscopy

After being cultured for 2 days, the cells were rinsed with phosphate-buffered saline (PBS) and fixed with methanol for 25 min at 4°C. They were incubated with monoclonal anti-phosphorylated neurofilament 200-kDa antibody (Sigma, MO, USA). After washing five times with PBS, the cells were treated with FITC-conjugated goat anti-mouse IgG (CHEMICON International Inc. CA, USA) and were then subjected to fluorescence microscopy.

### Cell proliferation assay

Cells (1.0 × 10^3^) were seeded in each well of a 96-well flat bottom culture plate and cultured for 1, 3 and 7 days. Cell proliferation was measured by Alamar Blue dye reduction assay (Trek Diagmpstic Systems, OH, USA). Briefly, Alamar Blue solution (10% final concentration vol/vol) was added to each well and the plate was incubated for 2h at 37°C. Dye reduction was measured in a Mithras LB940 microplate reader (Berthold Japan, Tokyo, Japan) at 530 nm excitation and at 590 nm emission. Cell proliferation rate was calculated as the fold increases, compared with the value at day 1.

### Statistics

All values are presented as the mean ± standard deviation of the mean (S. D.). All experiments were replicated at least three times. Data were analysed using Student's T-test with the Exel software package.

## Results

### Establishment of Neuro2a-AhR stable transfectant

**Figure 1:**
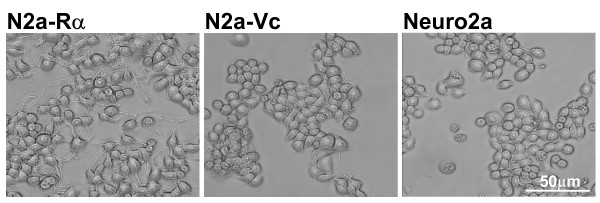
**Observation of N2a-Rα cells by phase contrast microscopy**. The parental Neuro2a cells (panel "Neuro2a") were transfected with rat AhR in the mammalian expression vector pcDNA4/V5-His and selected with zeocin. The morphology of the resultant transfectant is shown in panel "N2a-Rα ". The cells transfected with the vector without the insert are shown in panel "N2a-Vc". The N2a-Rα cells exhibited spontaneous neurite outgrowth. Note that no morphological differences in N2a-Vc were observed compared with the parental Neuro2a cells.

To investigate the function of AhR in developmental neurotoxicity, we attempted to establish Neuro2a-AhR transfectants. The murine neuroblastoma cell line, Neuro2a, was transfected with AhR cDNA in the expression vector (pcDNA4/V5-His), which includes the V5 epitope and the cytomegalovirus promoter. Seventy-five stably transfected clones were obtained after selection with zeocin. One of the clones, named N2a-Rα, was selected and used in a further series of experiments. Figure 1 shows the morphology of N2a-Rα, compared with control cells transfected with the intact vector (named N2a-Vc) and the parental Neuro2a cells (Neuro2a). Spontaneous neurite outgrowth was observed only in N2a-Rα, but not in N2a-Vc and Neuro2a. Most of the 75 transfectants exhibited similar morphology and displayed spontaneous neurite outgrowth.

### Transcriptional activation of exogenous AhR

**Figure 2:**
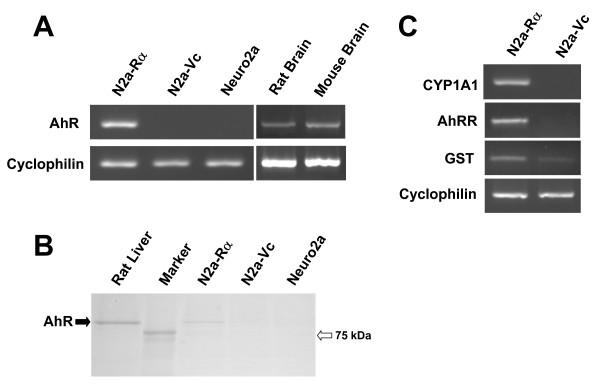
**Transcriptional activation of exogenous AhR in the N2a-Rα cells**. (A) AhR mRNA in N2a-Rα was detected by RT-PCR using primers specific for AhR. In N2a-Vc and Neuro2a, the primers amplified no products. The left panel shows that the primers amplified both mouse and rat AhR. Representative data are presented from triplicate experiments. (B) AhR protein (96kDa) in N2a-Rα was detected by Western blot analysis using a monoclonal antibody specific for AhR. In N2a-Vc and Neuro2a cells, no signals were detected. Representative data are presented from triplicate experiments. (C) The expression of CYP1A1, AhRR and GST mRNAs was detected in N2a-Rα cells. Since these genes were XRE-mediated genes, expression of these mRNAs are functional markers of AhR activation. Representative data are presented from triplicate experiments.

RT-PCR analysis was performed using mRNA isolated from transfectants to evaluate the expression of AhR mRNA with AhR-specific primers. The AhR primers were designed for rat AhR, but also specifically recognize and amplify mouse AhR under identical PCR conditions (Fig 2A). AhR mRNA was not detected in either the original Neuro2a cells or N2a-Vc cells transfected with the vector that did not contain the insert. On the other hand, AhR mRNA was clearly detected (Fig. 2A) in N2a-Rα. Since the primers for AhR amplify both rat and mouse AhR, these results indicate that N2a-Rα expressed AhR mRNA at a high level and that N2a-Vc expression of AhR was below the detection limit. The expression of AhR in N2a-Rα cells was reflected in the protein levels (Fig. 2B). Although immunoreactivity for AhR was not detected in the N2a-Vc and Neuro2a cells, the protein band corresponding to the molecular weight of AhR was clearly detected in N2a-Rα.

In order to confirm the transcriptional activity of the exogenous AhR in N2a-Rα, we analyzed CYP1A1, GST and AhRR (AhR repressor) mRNA expression as functional markers of AhR [8-11,29]. These genes were reported to be regulated by AhR binding to the XRE. The ligand-activated AhR binds to the XRE sequence in the promoter region of the CYP1A1 gene to activate its expression [30]. As such, CYP1A1, GST and AhRR mRNA would be induced if functional AhR is expressed in N2a-Rα. All mRNA species were detected in N2a-Rα cells with RT-PCR (Fig. 2C). These results suggest that the transfected AhR was expressed and functional in the N2a-Rα cells, even without the application of ligand to the culture medium.

**Figure 3:**
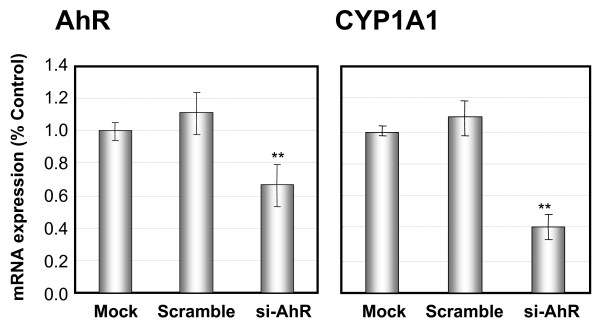
**Suppression of CYP1A1 mRNA expression by si-AhR**. These graphs demonstrate the repression of AhR and CYP1A1 mRNA expression by si-AhR transfection. N2a-Rα cells were transfected with AhR-targeted siRNA (si-AhR) or scramble siRNA. RNA was isolated from the cells transfected with siRNA and the expression levels were quantified by real-time quantitative PCR. AhR mRNA expression was effectively repressed by si-AhR (panel "AhR"). CYP1A1 mRNA expression was also decreased in cells transfected with si-AhR (panel "CYP1A1"). Scramble siRNA did not exhibit any effect on the mRNA expressions of AhR and CYP1A1 compared with Mock. The results are normalized to the expression level of cyclophilin mRNA and expressed relative to the value in the Mock. Values are presented as the mean of ± S. D. of four replicate determinations; ** p < 0.01 and *p < 0.05.

If the AhR stimulated the expression of XRE-mediated genes in N2a-Rα, such as CYP1A1, this expression would be attenuated by suppressing AhR activity. In this context, we attempted to suppress the AhR transcription using RNA interference. Two siRNAs (AhR and scramble, see "Methods" section) were transfected into N2a-Rα cells. Two days after transfection, the expression of AhR and CYP1A1 mRNA was measured by real-time quantitative PCR. As a result, AhR mRNA was decreased about 30% in N2a-Rα cells transfected with AhR siRNA, while the CYP1A1 mRNA level was decreased about 60% (Fig. 3). The expression levels of the AhR and CYP1A1 were not affected by the transfection of the scramble siRNA.

As stated above, the expression of both AhR and CYP1A1 were high in N2a-Rα, and were suppressed with siRNA against AhR. These results suggest that the activation of CYP1A1 was caused by over-expression of the AhR; specifically, the AhR expressed in N2a-Rα is functional as if activated by a ligand. The excess mRNA expressed in N2a-Rα cells might be sufficient for substituting the ligand-activated AhR.

### Properties of N2a-Rα neurites

**Figure 4:**
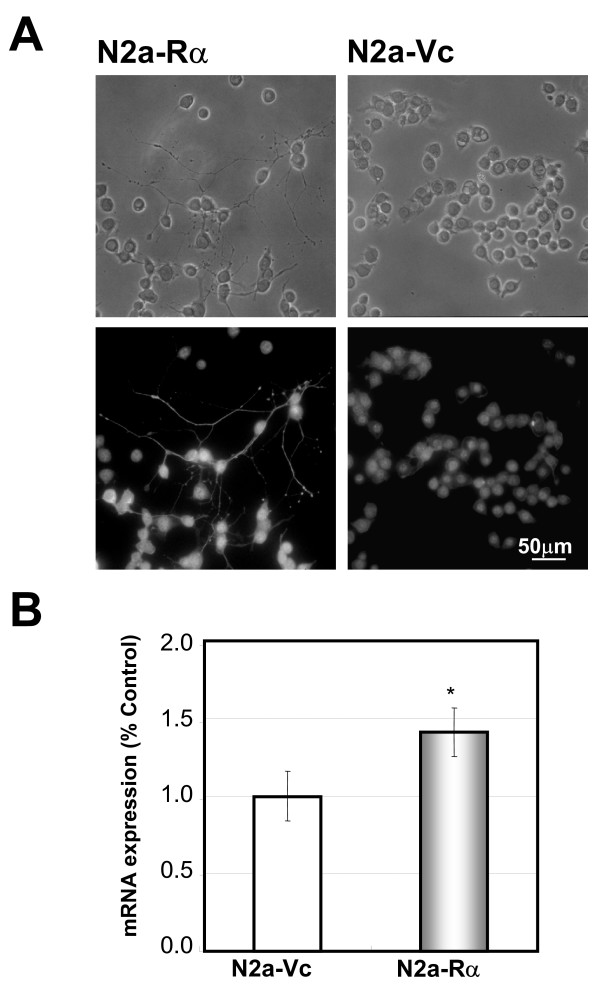
**Properties of neurites elongated from N2a-Rα cells**. (A) Immunochemical staining for phosphorylated NF-H was used as an axonal marker in N2a-Rα and N2a-Vc cells. Cells cultured for two-days were stained using the anti-phosphorylated neurofilament 200kDa (NF-H) monoclonal antibody and the FITC-conjugated goat anti-mouse IgG antibody (upper two panels). The lower panels are bright-field images of the upper panels. It should be noted that the outgrowths on the N2a-Rα cells were clearly stained with the anti NF-H antibody (panel "N2a-Rα "). (B) The quantity of cdc42 mRNA was determined in N2a-Rα and N2a-Vc cells with real-time PCR using primers specific for cdc42. A high level of expression of cdc42 mRNA was observed in N2a-Rα compared with the N2a-Vc. The results are normalized to the expression level of cyclophilin mRNA and expressed relative to the value of N2a-Vc. Values are presented as the mean ± S. D. of four replicate determinations; **p < 0.01 and *p < 0.05

Neurite elongation of the N2a-Rα cells implied their neuronal differentiation. Further analyses were performed to investigate the properties of the neurites that spontaneously elongated from N2a-Rα cells. The N2a-Rα cells (Fig. 1) were stained immunocytochemically with anti-cytoskeletal molecule antibodies. We selected the phosphorylated high molecular weight form (NF-H) as a mature axon marker [24,31]. Phosphorylated NF-H is localized in mature axons, but not in dendrites. N2a-Rα cells and N2a-Vc cells were immunostained with monoclonal anti-phosphorylated neurofilament 200-kDa antibody and FITC-conjugated goat anti-mouse IgG. The neurites exhibited positive immunostaining for phosphorylated NF-H (Fig. 4A). On the other hand, no fluorescent signal was observed after staining with MAP2a and 2b, which are markers of dendrites (data not shown) [32].

The expression of cdc42 mRNA in N2a-Rα was assessed using real-time PCR, and compared to N2a-Vc. The expression of cdc42 mRNA in N2a-Rα was approximately 35% higher than that of N2a-Vc (Fig. 4B). cdc42 is a member of the Rho family of small GTPases and is involved in cytoskeletal reorganization and the subsequent morphological changes in various cell types [33,34]. In PC12 cells, which have been used as a model system for neural differentiation and neurite outgrowth, expression of cdc42 has been reported to promote neurite outgrowth [35]. Recent evidence has supported that cdc42 is essential for the normal development of axons and plays a critical role in axon navigation [36]. Therefore the increased expression of cdc42 in N2a-Rα suggests that the processes elongated from N2a-Rα might be axons.

**Figure 5:**
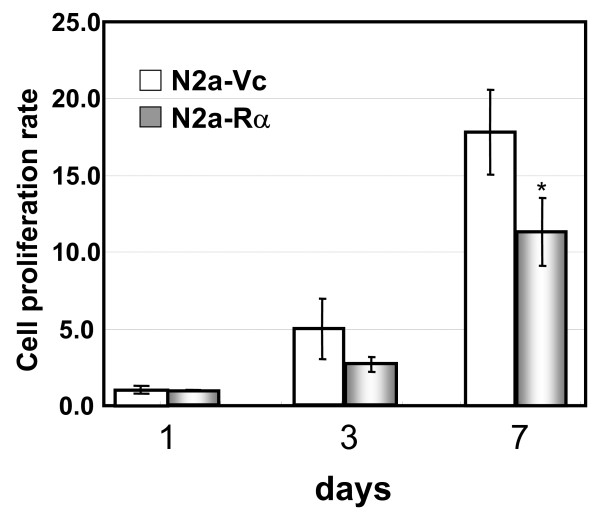
**Cell proliferation of N2a-Rα cells**. 1.0 × 10^3 ^cells were seeded in 96-well plates. After 1, 3 and 7 days of culture *in vitro*, cell proliferation in each well was measured by Alamar Blue dye reduction assay. Values are presented as the mean ± S. D. of four replicate determinations; *p < 0.05

In addition, we measured cell proliferation rates by Alamar Blue dye reduction assay based on the resazurin reduction test [37]. Cell proliferation was measured by quantitation of the reduction of the intracellular environment. The internal environment of the proliferating cell was attenuated compared with that of non-proliferating cells. Specifically, the ratios of NADP/NADPH, FADH/FAD, FMNH/FMN, and NADH/NAD, increase during proliferation. Alamar blue, which can be reduced by these metabolic intermediates, is useful in monitoring cell proliferation [38,39]. If N2a-Rα cells differentiate into neurons, cell growth would be reduced. The proliferation of N2a-Rα cells decreased by approximately 40% after 7 days, compared to N2a-Vc (Fig. 5). We also analysed the possibility of apoptosis in N2a-Rα cells. After 7-day culture, caspase-3/7 activities were not far differed from N2a-Vc (data not shown).

Together these results suggest that the N2a-Rα neurites may be considered to exhibit axon-like properties, and the morphological changes of N2a-Rα might be consistent with neuronal differentiation.

### Effect of AhR activation on catecholaminergic differentiation

**Figure 6:**
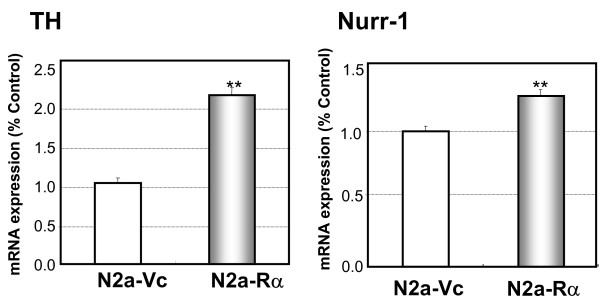
**Expression of catecholaminergic neuronal markers in N2a-Rα cells**. The expression of marker of catecholaminergic neuron, namely TH and Nurr is examined. The expression of TH mRNA and Nurr mRNA in the N2a-Rα cells and N2a-Vc cells were measured by RT-PCR with specific primers for TH and Nurr. Approximately twice the amount of TH mRNA was detected in the N2a-Rα cells compared with the N2a-Vc cells. Nurr mRNA was also detected significantly. Data were normalized to the expression level of cyclophilin mRNA and presented relative to the value of N2a-Vc. Values are presented as the mean ± S. D. of four replicate determinations; **p < 0.01 and *p < 0.05

It has been demonstrated that N2a-Rα cells exhibit neuron-like morphology with neurites that display axon-like properties. To further characterize the neuronal differentiation of N2a-Rα, we examined neurotransmitter production, which is characteristic of functional neurons. We assessed the expression of tyrosine hydroxylase (TH) and Nurr-1. TH is the rate-limiting enzyme in the production of neurotransmitters and is considered an adequate marker of catecholaminergic neurons [40,41]. Nurr-1 regulates the expressions of TH mRNA [42]. It has been reported that over-expression of Nurr-1 induced neurite elongation and led to differentiation of neural stem cells to dopaminergic neurons [43-45]. The level of TH mRNAs in the N2a-Rα and N2a-Vc cells was evaluated using RT-PCR. The amount of TH mRNA in the N2a-Rα cells was approximately twice that in the N2a-Vc cells (Fig. 6A). The level of Nurr-1 mRNA in N2a-Rα and N2a-Vc cells was also examined and higher levels were detected in N2a-Rα compared to N2a-Vc (Fig. 6B). This is supporting the neuronal differentiation of N2a-Rα, including neurotransmitter production. It is very likely that N2a-Rα cells differentiated into catecholaminergic neuron-like cells, triggered by the over-expression of AhR.

## Discussion

Activation of the AhR by dioxins results in the induction of xenobiotic metabolism and successive toxic responses, including hepatocellular damage, immune system disorders, reproductive system disorders, teratogenesis and carcinogenesis [46-48]. In the immune system, activated AhR suppresses a variety of B-cell mediated responses through aberrations in bone marrow B-cell development [49]. In the reproductive system, activated AhR causes depletion of fetal oocytes by apoptosis [50,51]. In response to unknown developmental cues, in addition to those stated above, the AhR can influence normal development.

In the nervous system, dioxins and related compounds are suspected to cause neurological disruption in human and experimental animal offspring following perinatal exposure during development and growth [52,53]. The perinatal period is a critical time for brain development with regard to formation of the neural network [54,55]. The dioxin receptor, AhR, is likely to participate in the developmental neurotoxicity of dioxins.

AhR-mediated pathways exist to mediate the effects of TCDD in many organs such as the liver, reproductive organs or immune tissues. [5,6]. However, in the brain, the mechanism involved in AhR-mediated transcription has been less well-elucidated. Therefore, we attempted to analyze the participation of AhR activation following exposure of the developing neuron to TCDD, by producing and using N2a-Rα cells, which were stably transfected with AhR cDNA in an undifferentiated neuroblastoma cell line, Neuro2a.

We have shown that the AhR is also functional in N2aRα cells even in the absence of ligand. This is based on the finding that the increase of CYP1A1 mRNA expression caused by over-expression of exogenous AhR was clearly suppressed by repression of the AhR with siRNA. There have been several other examples in which transfected AhR has been found to be active in the host cells [56,57], although it remains unclear why ligand-dependent AhR is activated under conditions in the absence of either ligand or TCDD.

The possible underlying mechanisms of AhR responses have been discussed previously. In many cell models, the AhR has been shown to stimulate growth arrest and apoptosis [58]. For example, in hepatocytes AhR activation promotes apoptosis in response to Fas stimulation [59]. However, AhR-induced apoptosis has not been reported in neurons, excluding the dorsal midbrain of zebrafish [60]. In this study, N2a-Rα did not give rise to cell death or apoptosis. N2a-Rα cells exhibited neuron-like features morphologically and functionally. Neurites were elongated from N2a-Rα, despite the fact that the transfectant possessed an intact vector (N2a-Vc) and the parental Neuro2a cells (Neuro2a) exhibited no process elongation. From a functional perspective, N2a-Rα cells exhibited elevation of TH mRNA, which is a selective marker of catecholaminergic neurons. These observations in N2a-Rα cells suggest that AhR over-activation in undifferentiated neurons triggered catecholaminergic neural differentiation, but not cell death. We hypothesized that the diversity of AhR toxicity may depend on AhR functions that are specific to each tissue or cell, although the physiological role of the AhR was not fully investigated.

Despite the fact that little evidence supporting the physiological function of the AhR in neural cells has been found so far, it has been reported that AhR is a member of the bHLH family transcription factor family. Although the AhR is an orphan receptor whose function has not been revealed, it is helpful for understanding AhR function to consider the relationships between AhR and other members of the bHLH family. Members of the bHLH family form a molecular-signaling network related to neural development and differentiation. For example, Hes-1 (the Hairy and Enhancer of Split homolog-1) and Hes-5 down-regulate Mash-1 and Math-1, respectively. Mash-1 and Math-1 subsequently down-regulate NeuroD and Math-2, and finally regulate the expression of nerve-specific genes [61]. In this manner, Hes-1 is known to be a key modulator in controlling neural proliferation and differentiation, but its function differs depending on the type of cells. On one hand, the AhR up-regulates Hes-1 through the XRE binding [62]. On the other hand, Hes-1 restrains neuronal differentiation and also inhibits cell proliferation in PC12 cells [63]. Although the function of Hes-1 in Neuro2a remains unknown, we hypothesize that the AhR might mediate neural development and differentiation through Hes-1 in the same manner as other members of the bHLH family gene network.

Consequently, in developing neurons, irregular or excessive activation of AhR possibly causes disrupted neural development rather than cell death. In the brain, TCDD exposure during the perinatal period may affect XRE-regulated gene expression through AhR activation and disrupt the differentiation of neurons, which are strictly regulated for developing brain formation

## Conclusion

In this study, we have shown that over-expression of AhR caused neural differentiation of Neuro2a cells. Neruo2a cells transfected with AhR, named N2a-Rα, may mimic the neurons forming the neural network during the perinatal stages, expressing AhR in the presence of the ligand. The AhR is a major participant in the expression of neurotoxicity and plays a role in the structural and functional development of neural cells. We hypothesized that perinatal exposure to dioxins may disrupt the strict regulation of brain formation with irregular differentiation rather than cell death. In consequence, the decline of neural function based on excess differentiation of neurons by AhR activation may play an important role in the developmental neurotoxicity of dioxins.

## Abbreviations

AhR, aryl hydrocarbon receptor

XRE, xenobiotic responsive element

TH, tyrosine hydroxylase

bHLH-PAS, basic helix-loop-helix/Per-Arnt-Sim

TCDD, 2, 3, 7, 8, -tetrachlorodibezo-*p*-dioxin

CYP, cytochrome P-450

FITC, fluorescein isothiocyanate

## Competing interests

The author(s) declare that they have no competing interests.

## Authors' contributions

EA designed the study and performed all experiments. SY was responsible for the cloning and maintenance of all cell strains constructed. MIS collaborated on experimental design, participated in data analysis, and wrote the manuscript. All authors read and approved the final manuscript.
